# Self-efficacy and enjoyment of physical activity in children: factorial validity of two pictorial scales

**DOI:** 10.7717/peerj.7402

**Published:** 2019-07-29

**Authors:** Milena Morano, Laura Bortoli, Montse C. Ruiz, Francesca Vitali, Claudio Robazza

**Affiliations:** 1 Parisi-De Sanctis Institute, MIUR (Italian Ministry of Education, University and Research), Foggia, Italy; 2 School of Medicine and Health Sciences, “G. d’Annunzio” University of Chieti-Pescara, Chieti, Italy; 3 BIND—Behavioral Imaging and Neural Dynamics Center, Department of Medicine and Aging Sciences, “G. d’Annunzio” University of Chieti-Pescara, Chieti, Italy; 4 Faculty of Sport and Health Sciences, University of Jyväskylä, Jyväskylä, Finland; 5 Department of Neurosciences, Biomedicine, and Movement, University of Verona, Verona, Italy

**Keywords:** Physical education, Primary school, Psychological constructs, Assessment, Confirmatory factor analysis

## Abstract

**Background:**

Self-efficacy and enjoyment are two main constructs proposed within many motivational theories in any human endeavor, sport and physical activity included.

**Methods:**

The purpose of this study was to examine the factor structure of two pictorial scales measuring self-efficacy and enjoyment levels in a sample of 14,035 Italian schoolchildren (7,075 boys and 6,960 girls, 6- to 7-year-olds). An important feature of the two scales is that they are in a pictorial format in order to prompt a straightforward understanding in children. The whole sample was randomly split in two subsamples according to gender and age and the factor structure of the measures was examined across subsamples.

**Results:**

Data were subjected to confirmatory factor analysis, which yielded satisfactory fit indices on the measures of both subsamples. Overall findings supported the single factor structure of the scales, which can be easily administered to 6- to 7-year-old children to assess two relevant psychological constructs in physical education.

## Introduction

Self-efficacy and enjoyment are central mechanisms underlying motivated behaviours, such as sport and physical activity (see Bandura, 1997; Ryan & Deci, 2017). Self-efficacy is defined as an individual’s belief in their own capabilities to accomplish a task or succeed in specific situations; it is considered the cognitive mechanism that mediates information on personal capacities to successfully execute necessary courses of action in a specific domain (Bandura, 2001). In the context of physical education and sport, self-efficacy has been extensively examined (Feltz, Short & Sullivan, 2008) and identified as an important correlate of physical activity and fitness in mediating children achievement striving (Feltz, 1992; McAuley & Blissmer, 2000; Barnett et al., 2011). In fact, research evidence has shown physical self-efficacy to be both a determinant and a consequence of physical activity (McAuley, Peña & Jerome, 2001). Efficacy beliefs refer to judgments about the ability to accomplish a task, while previous positive experiences of accomplishments can enhance self-efficacy beliefs. Hence, an individual’s judgement of motor ability and skill levels can be considered an important factor in self-perception, because successful performance is associated with high self-efficacy (Moritz et al., 2000). Furthermore, physical performance and self-perceived physical fitness are positively related to perceived competence (Sollerhed et al., 2008), which has been shown to predict participation in physical activity (Bauman et al., 2012; Di Battista et al., 2018) and enjoyment (Cairney et al., 2012).

In the physical activity and sport domains, enjoyment is conceptualized as a positive affective response resulting from participation that reflects generalized feelings typically described as pleasure, liking, and fun (Scanlan & Simons, 1992). The experience of enjoyment during physical education classes is associated with enhanced intrinsic motivation, increased physical activity participation, and the adoption of active and healthy lifestyles (Wallhead & Buckworth, 2004; Dishman et al., 2005; Jaakkola et al., 2017; Bortoli et al., 2018; Vitali et al., 2019). Thus, understanding enjoyment motives and other variables known to influence physical activity levels, successful motor experiences, and improvement of physical fitness can help researchers and practitioners design more effective intervention strategies to promote healthy lifestyles among school-aged children.

In health psychology literature, physical self-efficacy and enjoyment are currently considered important correlates and determinants of physical activity and healthy behaviors in adults (Trost et al., 2002), children, and adolescents (Lubans, Foster & Biddle, 2008). To broaden our understanding of the mechanisms underlying the antecedents and consequences of these two variables, there is a need of valid and reliable measures in the assessment of people of all ages, children included.

Different measures have been developed to gauge self-efficacy in 8–12-year-old children. Some of them are often used in health research and refer to coping self-efficacy, which is the confidence in performing physical activity despite encountering social or environmental barriers (e.g., “I can be physically active even if I have to stay at home”; Bartholomew et al., 2006). Other measures refer to task self-efficacy, namely, the confidence in using specific motor capabilities or performing skills (e.g., “I am able do to very difficult exercises”; Colella et al., 2008).

Enjoyment in physical activity is often measured using the Physical Activity Enjoyment Scale (PACES; Kendzierski & DeCarlo, 1991). The PACES is a scale consisting of 16 bipolar items, originally developed to assess the extent to which individuals enjoy doing any given physical activity. Preliminary evidence of reliability and validity has been found with samples of university students. Motl et al. (2001) revised the PACES for use with young adolescent females. Moore et al. (2009) found the revised form of the PACES a valid measure of enjoyment of physical activity also in 8-year-old children.

De Civita et al. (2005) highlighted the need to pay special attention in developing assessment instruments adequately validated for young children. To maximize reliability and validity, the items need to be adapted to the individual developmental stage, level of emerging sense of self, cognitive capacity, and emotional awareness. Given the importance of assessing self-efficacy and enjoyment constructs in children, the purpose of this study was to develop two new short measures typified by a response pictorial format adequate for 6–7-year-olds, and to examine their factor structure. We also examined possible differences in self-efficacy and enjoyment by gender and age.

## Materials & Methods

### Participants

The sample consisted of 14,035 children aged 6 to 7 years (7,075 boys and 6,960 girls). The participants were drawn from about 800 mixed gender classes of primary schools located in a region in Central Italy. All classes were involved in a large project of physical activity named “At school of health: Increase in physical activity in the I and II classes of Primary School”. The main goal of the project was to prevent obesity and promote healthy lifestyles in children. Physical activity was conducted during customary lessons held by expert physical education teachers.

### Measures

#### Self-efficacy

Colella et al. (2008) developed a 6-item physical self-efficacy scale to assess perceived speed, strength, coordination, and fatigue in girls and boys ranging in age from 8 to 10 years. To render the scale more easily understandable by younger children (i.e., 6–7-year-olds) and to help them grasp the meaning of the items, we selected four statements and slightly modify the items by representing them with emoticons and pictograms (see Scales S1). Item scores ranged from 1, indicating low efficacy (e.g., “I run very slow”) to 4, representing high efficacy (e.g., “I run very fast”).

#### Enjoyment

Individuals’ enjoyment of physical activity was measured using four items selected from the 16-item Physical Activity Enjoyment Scale (PACES; Carraro, Young & Robazza, 2008), which was intended to gauge enjoyment of 11 to 19 years old students involved in physical education classes at school. The scale was slightly modified by anchoring the items to emoticons to render them easily understandable by children (see Scales S1). Item scores ranged from 1 (not at all) to 5 (very much).

For both scales, participants are required to think of themselves when playing or performing physical education exercises. They are then asked to indicate for each item the response that best represents their personal feelings.

### Procedure

Agreement to conduct the study was sought from the school headmasters after the purpose of the study had been explained to them. All the participants’ parents provided written informed consent with anonymity and confidentiality being assured for all the participants. Ethical approval for the study was obtained from the Health Department of the Abruzzo Region in reference to the Regional Prevention Plan 2014–2018 - Program 2, Action 2.

Assessment was conducted by a team of experts specifically instructed in the assessment procedure. The measures were administered in small groups of children at the end of physical education lessons and without the presence of the teachers. Children were presented with the scale, informed that there were no right or wrong responses, and assured that their answers were confidential. Before commencing the assessment, the researchers made sure that all children had a correct understanding of the instructions and items.

### Data analysis

Data were preliminarily examined for missing values, and 124 cases with missing data were deleted. To examine the factor structure of the measures, the whole sample was randomly split in two subsamples, which were homogeneous in terms of gender and age (see Data S2). Confirmatory factor analysis (CFA) was then conducted to assess the factorial validity of the scales across subsamples. Given that data distribution of both measures was negatively skewed, a robust diagonally weighted least squares (DWLS) estimation was used. In particular, model parameters, standard errors, and chi-square statistics robust to non-normality were estimated using the weighted least squares mean- and variance-adjusted method (WLSMV; Muthén & Muthén, 2017), which is appropriate for estimating CFA model parameters with ordered categorical variables (see Finney & DiStefano, 2013). Flora & Curran (2004) demonstrated that WLSMV produces accurate test statistics, parameter estimates, and standard errors of CFA models with sample sizes ranging from 100 to 1,000. Although WLSMV works well with relatively small sample sizes, according to Brown (2015) very skewed categorical indicators call for larger samples. This is a reason why we involved a very large sample of children in our study. The comparative fit index (CFI), the Tucker Lewis fit index (TLI), the root mean square error of approximation (RMSEA), and the standardized root mean square residual (SRMR) were examined. A good model fit is inferred when CFI and TLI values are close to .95, SRMR is lower than .08, and RMSEA is lower than .06 (Browne & Cudeck, 1993; Hu & Bentler, 1999; Schumacker & Lomax, 2004). All data analyses were conducted in M*plus* version 8.3 (Muthén & Muthén, 2017).

## Results

Descriptive statistics and fit indices of the self-efficacy scale and the enjoyment scale across subsamples, gender, and age are reported in Table 1. As can be seen, CFA results yielded satisfactory fit indices on both measures. CFA was also conducted on the measurement model (i.e., the two measures together) on the data of the whole sample. Good fit indices for this model were found: CFI = .987, TLI = .980, RMSEA = .039 (90% CI [.036–.042]), SRMR = .027, with standardized factor loadings ranging from .649 to .810. Correlation between self-efficacy scale and enjoyment latent factors was .540.

**Table 1:** Descriptive statistics, fit indices, and standardized factor loadings for self-efficacy and enjoyment scales across subsamples, gender, and age.

Subsample (*n*)	Gender, age	Scale	*M* ± *SD*	*χ*^2^ (*df* = 2)	CFI	TFI	SRMR	RMSEA (90% CI)	Factor loadings (min–max)
1 (1674)	Girls, 6 yrs.	Self-efficacy	3.39 ± 0.56	2.313	1.000	.999	.006	.010 (.000–.050)	.592–.785
		Enjoyment	4.78 ± 0.44	3.440	.999	.998	.010	.021 (.000–.057)	.706–.826
2 (1673)		Self-efficacy	3.47 ± 0.55	1.468	1.000	1.001	.005	.000 (.000–.044)	.686–.769
		Enjoyment	4.81 ± 0.39	9.672	.997	.992	.013	.048 (.021–.080)	.775–.850
1 (1696)	Boys, 6 yrs.	Self-efficacy	3.53 ± 0.49	7.438	.995	.984	.013	.040 (.012–.073)	.577–.736
		Enjoyment	4.78 ± 0.46	4.378	.999	.996	.010	.026 (.000–.061)	.676–.843
2 (1696)		Self-efficacy	3.58 ± 0.48	.085	1.000	1.005	.001	.000 (.000–.000)	.631–.703
		Enjoyment	4.80 ± 0.45	7.228	.998	.994	.009	.039 (.011–.072)	.788–.863
1 (1807)	Girls, 7 yrs.	Self-efficacy	3.42 ± 0.49	4.103	.999	.997	.008	.024 (.000–.058)	.635–.741
		Enjoyment	4.81 ± 0.33	20.353	.984	.953	.029	.071 (.045–.101)	.622–.804
2 (1806)		Self-efficacy	3.50 ± 0.48	15.956	.995	.984	.016	.062 (.036–.092)	.675–.802
		Enjoyment	4.82 ± 0.33	17.995	.993	.980	.020	.067 (.041–.096)	.741–.844
1 (1842)	Boys, 7 yrs.	Self-efficacy	3.53 ± 0.48	11.551	.995	.985	.014	.051 (.025–.081)	.670–.725
		Enjoyment	4.77 ± 0.42	4.081	.999	.997	.009	.024 (.000–.057)	.710–.828
2 (1841)		Self-efficacy	3.59 ± 0.44	16.623	.991	.973	.018	.063 (.037–.093)	.641–.758
		Enjoyment	4.80 ± 0.36	5.582	.998	.994	.011	.031 (.000–.063)	.718–.806

**Notes.**

*χ*^2^(*df*) chi-square (degrees of freedom) CFI comparative fit index TLI Tucker Lewis fit index SRMR standardized root mean square residual RMSEA root mean square error of approximation

Confirmatory factor analysis fit indexes are satisfactory for both measures across subsamples, gender, and age.

To examine possible gender and age differences on the total sample, mean scores of the two scales were transformed using the NEWX = 1/(K–X) formula proposed to adjust negatively skewed data (Tabachnick & Fidell, 2013), where the constant K was the largest score + 1. Multivariate analysis of variance (MANOVA) by gender and age on the transformed mean scores of the scales yielded significant results for gender, Wilks’ *λ* = .984, *F*(2, 14030) = 111.095, *p* < .001, ${\eta }_{\mathrm{p}}^{2}=.016$. Univariate follow-up showed significant differences on self-efficacy scores, *F*(1, 14031) = 177.166, *p* < .001, with boys scoring higher than girls. However, the effect size of the difference was small, Hedges’ *g* = .225 (90% CI [.197–.253]).

## Discussion

The aim of this study was to examine the factor structure of two pictorial scales measuring self-efficacy and enjoyment levels. Findings provided support for the internal consistency and factorial validity of a single-factor structure of the scales, which can be easily administered to 6–7-year-old children. Therefore, the results suggest that these scales can be used to assess self-efficacy and enjoyment of children involved in physical activities in school settings. Their use could promote additional research to examine, in particular, concurrent and predictive validity.

An important feature of the two scales is their pictorial format to make them easily understandable for children. The advantages of using pictorial scales initially emerged in clinical and experimental contexts interested in measuring perception of physical exertion in children and adolescents (Robertson et al., 2000). Indeed, perceived exertion scales for adults proved to be unreliable, because not equated with the cognitive development of children under the age of 9 and 10 years. In particular, 6–7-year-old children are at the start of the middle childhood stage, a developmental phase of cognitive capacities, physical self-perception, and emotional awareness. At this stage, children are unable to reliably report their perceptions and feelings by assigning numbers to words or phrases. They may also find difficult to understand words that do not belong to their current vocabulary (Williams, Eston & Furlong, 1994). For these reasons, Robertson et al. (2000) developed the Children’s OMNI Scale of Perceived Exertion, a perceived exertion scale specifically designed for use with children. The scale contains both pictorial and verbal descriptors representing a cyclist on a slope, positioned along a numerical scale ranging from 0 (not tired at all) to 10 (very, very tired). The term OMNI is a contraction of the word omnibus, to indicate a scale with broadly generalizable measurement properties. Different OMNI Scales were later developed to assess exertional perceptions of children engaged in other dynamic exercise modes, such as walking or running (Utter et al., 2002; for other examples of OMNI Scales, see Heyward & Gibson, 2014; Armstrong & Van Mechelen, 2017).

In contrast to the single item format of the OMNI Scales, Coulter & Woods (2011) used a pictorial style, self-report measure comprised of six items to examine children’s active behaviors out of school and their enjoyment in physical activities they partake in. However, the factor structure of the scale was not examined. In a more detailed perspective, Barnett et al. (2016) developed a pictorial instrument (PMSC; Pictorial scale for Perceived Movement Skill Competence) for children aged 4–5 years to gauge, with the help of an adult, their perception of six locomotor and six object control skills based on the Ulrich’s (2000) Test of Gross Motor Development (TGMD-2).

Drawing on previous studies using pictorial scales, we developed two new short, self-assessment pictorial scales measuring self-efficacy and enjoyment to be easily and quickly administered to children. These scales contained selected and adapted items from the Colella et al.’s (2008) physical self-efficacy scale for children and the Carraro, Young & Robazza’s (2008) Physical Activity Enjoyment Scale. Item responses were then transformed into a pictorial format to make them more “child friendly”. CFA yielded satisfactory fit indices across gender and age, thereby suggesting that the two scales can reliably gauge self-efficacy and enjoyment of physical activity in children. The negatively skewed distribution of scores in both measures indicates a general perception of high self-efficacy and enjoyment levels, which is desirable in children engaged in physical activity. Therefore, low scores on one or both scales may reveal individual issues related to the participation in physical tasks and suggest a need for appropriate interventions.

In a review of potential mediators of children’s physical activity, Brown, Hume & ChinAPaw (2009) underlined the need for future research examining the psychometric properties of measures of potential mediators in different study samples to ensure that appropriate, valid, and reliable instruments are used. More clearly identified associations between hypothesized mediators and physical activity can facilitate the development of more effective interventions. Physical activity is considered one of the most important factors to successfully prevent or treat childhood overweight and obesity (World Health Organization, 2016), and interventions are often targeting young children, even preschool children (Ward et al., 2016). Our scales may contribute to this call for use of appropriate instruments in the assessment of relevant constructs in physical activity settings, adequately validated in the specific population of interest.

For applied purposes, assessing self-efficacy and enjoyment can enable teachers to early identify children with negative attitudes toward physical activities. Most children like moving and playing, which are essential components of their development. Through active play children not only refine their physical abilities and learn motor skills, but also develop social relationships, self-confidence, and creativity (Truelove, Vanderloo & Tucker, 2017). Therefore, teachers should pay special attention to less active children, and find specific goals and strategies to enhance their intrinsic motivation. Moreover, valid measures would enable teachers to assess the effectiveness of physical activity programs aimed at enhancing children’s interest and motivation.

## Conclusions

Self-efficacy and enjoyment have important consequences on individuals’ quality of life (Kahneman, Diener & Schwarz, 1999; Morano et al., 2016), which is a multidimensional construct that reflects one’s perceptions of fitness, life satisfaction, and wellbeing (Bowling, 2001). Enhancing children enjoyment for physical education and their actual and perceived physical abilities are expected to stimulate the adoption of an active lifestyle and improve health-related quality of life (Vitali et al., 2019). Given the importance of the two constructs, we have developed two new short pictorial scales, very easy to administer, and adequate for 6–7-year-olds. Of note, the two scales do not require a direct assistance from an adult for the assessment of children.

A limitation of this study is that we only examined the factor structure of the two scales. Additional research is necessary to establish the validity and reliability of these scales in physical education and different physical activity domains, including sport and leisure. Further validation is also needed to take account of a range of variables, such as age, gender, physical abilities, body mass index, and culture (Crocker, Bouffard & Gessaroli, 1995). Taken together, our study findings provide initial evidence for the use of the two pictorial scales with 6–7-year-old children in physical education contexts.

##  Supplemental Information

10.7717/peerj.7402/supp-1 Supplemental Information 1 Raw data

10.7717/peerj.7402/supp-2 Supplemental Information 2 The physical self-efficacy scale and the physical activity enjoyment scale for children

## Additional Information and Declarations

### Data Availability

The following information was supplied regarding data availability: The raw data are available as a Supplemental File.
