# Supplementary material for: Self-efficacy and enjoyment of physical activity in children: factorial validity of two pictorial scales
Source: PeerJ. 2019 Jul 29;7:e7402. doi: 10.7717/peerj.7402 (PMC6673428; doi:10.7717/peerj.7402)
Supplement: Supplemental Information 2 [file peerj-07-7402-s002.pdf]

Supplemental scales S1:
The physical self-efficacy scale and the physical activity enjoyment scale for children

WHEN I PERFORM PHYSICAL ACTIVITY AT SCHOOL:

	1	2	3	4
1	I run very slowly 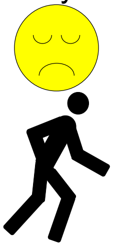	I run slowly 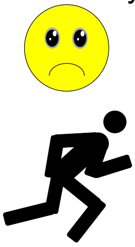	I run fast 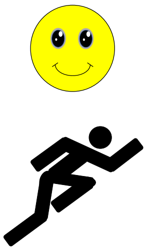	I run very fast 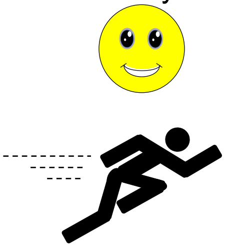
2	I am able to do very easy exercises only 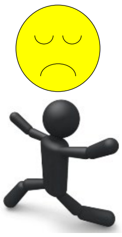	I am able to do easy exercises only 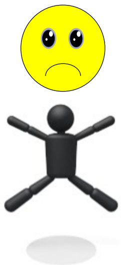	I am able to do difficult exercises 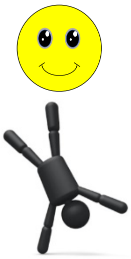	I am able to do very difficult exercises 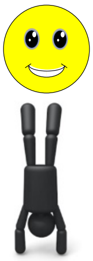
3	My muscles are very weak 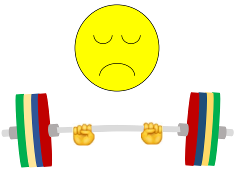	My muscles are weak 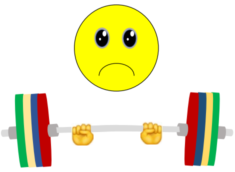	My muscles are strong 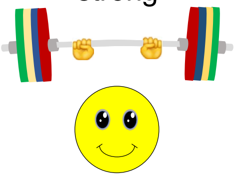	My muscles are very strong 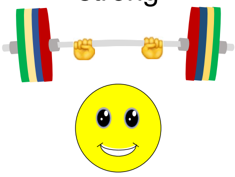
4	I feel very tired when I move 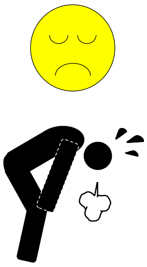	I feel tired when I move 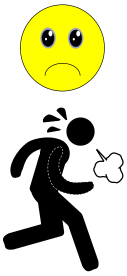	I don't feel tired when I move 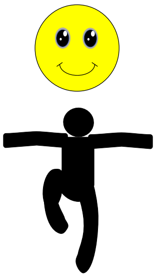	I don't feel tired at all when I move 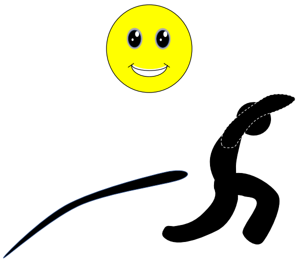

	Not at all 1	Very little 2	Little 3	Much 4	Very much 5
1. I enjoy it	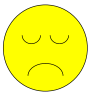	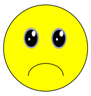	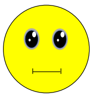	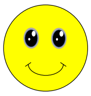	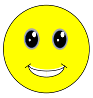
2. I like it	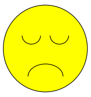	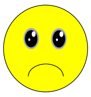	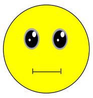	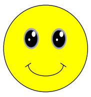	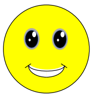
3. It gives me energy	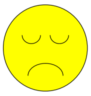	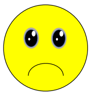	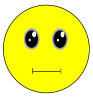	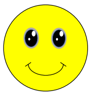	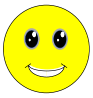
4. It makes me feel well	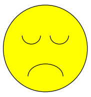	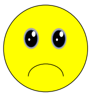	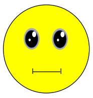	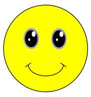	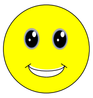

Italian version

QUANDO FACCIO ATTIVITÀ MOTORIE A SCUOLA:

	1	2	3	4
1	Corro pianissimo 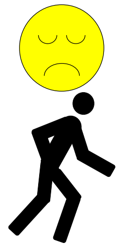	Corro piano 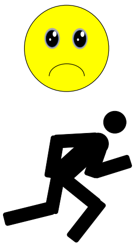	Corro veloce 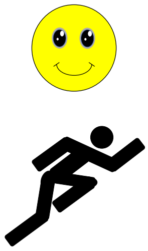	Corro velocissimo 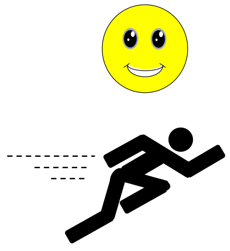
2	Sono capace di fare solo esercizi facilissimi 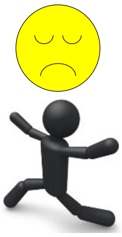	Sono capace di fare solo esercizi facili 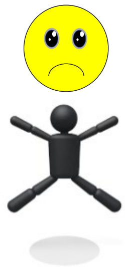	Sono capace di fare esercizi difficili 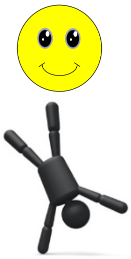	Sono capace di fare esercizi difficilissimi 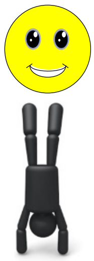
3	I miei muscoli sono debolissimi 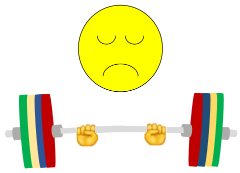	I miei muscoli sono deboli 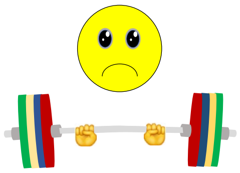	I miei muscoli sono forti 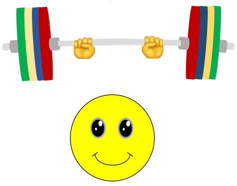	I miei muscoli sono fortissimi 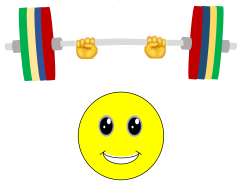
4	Faccio molta fatica quando mi muovo 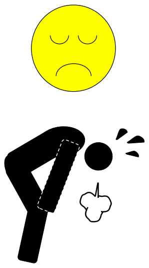	Faccio fatica quando mi muovo 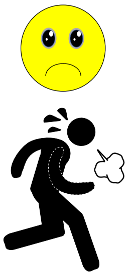	Non faccio fatica quando mi muovo 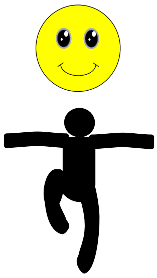	Non faccio proprio nessuna fatica quando mi muovo 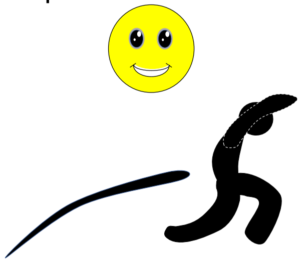

	Per nulla 1	Pochissimo 2	Poco 3	Molto 4	Moltissimo 5
1. Mi diverto					
2. Mi piace					
3. Mi dà energia					
4. Mi fa sentire bene					
